# Development by Genetic Immunization of Monovalent Antibodies Against Human Vasoactive Intestinal Peptide Receptor 1 (VPAC1), New Innovative, and Versatile Tools to Study VPAC1 Receptor Function

**DOI:** 10.3389/fendo.2018.00153

**Published:** 2018-04-05

**Authors:** Xavier Peyrassol, Toon Laeremans, Vannessa Lahura, Maja Debulpaep, Hassan El Hassan, Jan Steyaert, Marc Parmentier, Ingrid Langer

**Affiliations:** ^1^Institut de Recherche Interdisciplinaire en Biologie Humaine et Moléculaire (IRIBHM), Université libre de Bruxelles, Brussels, Belgium; ^2^Structural Biology Brussels, Vrije Universiteit Brussel (VUB), Brussels, Belgium; ^3^Structural Biology Research Center, VIB, Brussels, Belgium; ^4^Confo Therapeutics, Zwijnaarde, Belgium; ^5^Welbio, Université libre de Bruxelles, Brussels, Belgium

**Keywords:** vasoactive intestinal peptide, vasoactive intestinal polypeptide receptor, vasoactive intestinal peptide receptor 1, G protein-coupled receptor, monovalent antibodies, llama, DNA immunization

## Abstract

Multi-membrane spanning proteins, such as G protein-coupled receptors (GPCRs) and ion channels, are extremely difficult to purify as native proteins. Consequently, the generation of antibodies that recognize the native conformation can be challenging. By combining genetic immunization, phage display, and biopanning, we identified a panel of monovalent antibodies (nanobodies) targeting the vasoactive intestinal peptide receptor 1 (VPAC1) receptor. The nine unique nanobodies that were classified into four different families based on their CDR3 amino acid sequence and length, were highly specific for the human receptor and bind VPAC1 with moderate affinity. They all recognize a similar epitope localized in the extracellular *N*-terminal domain of the receptor and distinct from the orthosteric binding site. In agreement with binding studies, which showed that the nanobodies did not interfere with VIP binding, all nanobodies were devoid of any functional properties. However, we observed that the binding of two nanobodies was slightly increased in the presence of VPAC1 agonists [vasoactive intestinal polypeptide (VIP) and pituitary adenylate cyclase-activating polypeptide-27 (PACAP-27)], but decreased in the presence of VPAC1 antagonist. As no evidence of allosteric activity was seen in VIP binding studies nor in functional assays, it is, therefore, possible that the two nanobodies may behave as very weak allosteric modulators of VPAC1, detectable only in some sensitive settings, but not in others. We demonstrated that the fluorescently labeled nanobodies detect VPAC1 on the surface of human leukocytes as efficiently as a reference mouse monoclonal antibody. We also developed a protocol allowing efficient detection of VPAC1 by immunohistochemistry in paraffin-embedded human gastrointestinal tissue sections. Thus, these nanobodies constitute new original tools to further investigate the role of VPAC1 in physiological and pathological conditions.

## Introduction

The vasoactive intestinal peptide receptor 1 (VPAC1) is a member of the family B of G protein-coupled receptors (GPCRs), which includes VPAC2, PAC1, secretin, glucagon, GLP-1 and -2, calcitonin, GIP, CRF-1 and -2, and PTH receptors. The endogenous ligands of VPAC1 receptor are vasoactive intestinal polypeptide (VIP) and pituitary adenylate cyclase-activating polypeptide (PACAP). VPAC1 and VPAC2 receptors have similar high affinities for VIP and PACAP, whereas PAC1 receptor exhibits much lower affinity for VIP than for PACAP (1, 2). The two neuropeptides contribute to the regulation of intestinal motility and secretion, exocrine and endocrine secretions, and homeostasis of the immune system. Therefore, VIP and PACAP constitute potential therapeutic agents for the treatment of several diseases, such as chronic inflammation, neurodegenerative diseases, and cancer. However, the major drawbacks of their use are their high sensitivity to degradation by proteases and their ability to interact at high affinity with different receptors (1–3).

Over the past years, the implementation of biologics as therapeutics has gained considerable attention. Particularly, monoclonal antibodies (mAbs) became attractive therapeutics. They are highly selective and tend, therefore, to display fewer off-target toxicity issues, and are characterized by extended half-lives compared with small molecules. There are currently around 60 mAbs on the market, but only one GPCR-targeting antibody has been approved recently in Japan (4, 5). This can partly be explained by the fact that the generation of mAbs recognizing the native conformation of GPCRs is a challenging task, as they are extremely difficult to purify in their native conformation compared to soluble proteins. The most common approach is thus to immunize animals with synthetic peptides derived from the receptor and link them to larger, highly immunogenic carrier proteins. The resulting antibodies have, however, unpredictable specificity, affinity, and pharmacological properties, necessitating considerable screening efforts to identify good therapeutic candidates. They tend also to recognize linear epitopes, by increasing the risk of cross-reactivity with unrelated proteins and off-target toxicity (4–6).

Camelidae produce, in addition to conventional antibodies, a special type of antibodies devoid of light chains, which are called heavy chain only antibodies (7). Antigens are recognized through the 15 kDa *N*-terminal variable domain (VHH) of the heavy chain, which is frequently referred to as nanobody. For several years, nanobodies have generated a growing interest because of their potential advantages over conventional antibodies. Nanobodies were reported to display low immunogenicity, high solubility, superior stability, and greater tissue penetration. Their smaller paratope gives them access to cavities on the surface of proteins, epitopes usually not recognized by conventional antibodies, or engineered Fab and scFv fragments. Moreover, most nanobodies developed so far are able to modulate the function of their target (8, 9). Finally, they are easier and cheaper to produce in microbial expression hosts (7). Thus, nanobodies combine the advantages of both small molecules and mAbs and constitute new research tools to study the distribution, the pharmacology, and the role of GPCRs in physiological and pathological conditions. They may also serve as innovative tools as diagnostics and, potentially, as therapeutics (7, 8).

In this study, we have generated, using genetic immunization and phage display, a panel of nanobodies recognizing the VPAC1 receptor. These nanobodies are specific for the human receptor and do not recognize the closely related human VPAC2. They bind VPAC1 with moderate affinity, and their binding site, localized in the extracellular *N*-terminal domain of the receptor, is distinct from the orthosteric VIP binding site. Following fluorescent labeling, they detect native VPAC1 on human leukocytes in FACS experiments. We also developed a protocol allowing efficient detection of VPAC1 in paraffin-embedded human tissue sections by immunohistochemistry. These nanobodies thus constitute innovative tools to study the distribution and the role of VPAC1.

## Materials and Methods

### Cell Lines

CHO-WTA11 cells, co-expressing apoaequorin and Gα16, were cultured in Ham’s F12 medium (Thermo Fisher Scientific) supplemented with 10% fetal bovine serum (FBS, Thermo Fisher Scientific), 100 U/ml penicillin, 100 µg/ml streptomycin (Thermo Fisher Scientific), and 250 µg/ml zeocin (InvivoGen) at 37°C with a constant supply of 5% CO_2_. The cell lines expressing chimeric VPAC1/VPAC2 receptors, kindly provided by Magali Waelbroeck (ULB, Belgium), were described previously (10). To obtain the cell line expressing human VPAC1 fused at its *N*-terminus with a FLAG-tag, the FLAG coding sequence was inserted in frame at the 3′ end of the signal peptide sequence of VPAC1 cDNA in a homemade bicistronic vector (pCDneo). The resulting plasmid (2 µg) was transfected in CHO-WTA11 cells using FuGENE six transfection reagent (Roche) according to the manufacturer’s instructions. Selection of stably transfected cells was carried out in culture medium supplemented with 250 µg/ml G418 (InvivoGen). After 10–15 days of selection, individual clones were transferred to 24-well plates, grown to confluence, trypsinized, and further expanded in 6-well plates. The identification of cell clones expressing the VPAC1-FLAG fusion was performed by FACS using a rabbit polyclonal anti-FLAG antibody (CellSignaling; 1/50). A similar protocol was used to obtain the cell lines expressing human VPAC2, cloned in pCDneo. The identification of cell clones expressing VPAC2 was performed by FACS using a mouse monoclonal anti-VPAC2 antibody (11); (1/100).

Human embryonic kidney cells (HEK-293T) were cultured in Dulbecco’s modified Eagle’s medium (Thermo Fisher Scientific) supplemented with 10% FBS, 100 U/ml penicillin, and 100 µg/ml streptomycin at 37°C with a constant supply of 5% of CO_2_. HEK-293T was transiently transfected by the polyethylenimine (PEI) method. Briefly, 10^6^ cells were transfected with 2.5 µg of plasmids coding for receptors mixed with 1 mg/ml PEI 25,000 MW (Polysciences) in Dulbecco’s modified Eagle’s medium supplemented with 100 U/ml penicillin and 100 µg/ml streptomycin. The next day, the medium was replaced by medium containing 10% FBS. Cells were analyzed by FACS 48 h post-transfection. The plasmid coding for rat VPAC1 was a gift from E.M. Lutz (University of Strathclyde, UK).

### Genetic Immunization and Immune Repertoire Cloning

Camelid single domain antibody fragments (VHHs, nanobodies) specific for human VPAC1 were identified from immunoglobulin repertoires of genetically immunized llamas following a prime-boost regime (12). The human VPAC1 coding sequence was cloned into the mammalian expression vector pCDneo and endotoxin-free plasmid DNA was prepared using the EndoFree Giga kit (Qiagen) according to the manufacturer’s instructions. Two llamas (*Llama glama*) were immunized by intradermal administration of plasmid DNA (2 mg) at days 0, 14, 28, and 42 using a Dermojet device (Akra Dermojet). Twenty-one days after the last DNA immunization, 2 × 10^7^ CHO cells overexpressing VPAC1-FLAG and previously incubated during 1 h at 4°C with 3 µM VIP (Bachem) were injected subcutaneously. The VPAC1-specific serum conversion was monitored by flow cytometry during immunization (13). Four days after the fourth DNA vaccination and 4 days after the cell boost, 100 ml blood samples were collected from each llama and peripheral blood lymphocytes (PBLs) were prepared by Ficoll-Paque Plus density gradient centrifugation according to the manufacturer’s instructions (LeucoSep tubes, Greiner Bio-One). Total RNA was extracted from the PBLs as described by Chomczynski and Sacchi (14) and stored as ethanol precipitate at −80°C. First strand cDNA synthesis was performed using hexanucleotide random priming and the SuperScript III First-Strand Synthesis System (Thermo Fisher Scientific) according to the manufacturer’s instructions. The nanobody repertoire was amplified and cloned in pXAP100 with minor modifications of the method described (15). pXAP100 is a pMESy4 derivative allowing expression of SfiI/BstEII cloned *C*-terminally His6-cMyc-tagged nanobodies. For the nested PCR, the 5′ primer was adapted to contain the SfiI restriction site.

### Identification of Nanobodies Recognizing Native Extracellular VPAC1 Epitopes

Nanobody-displaying bacteriophage particles were produced according to standard protocols following helper phage superinfection (15). VPAC1-specific phages were enriched by two methods of biopanning using CHO cells expressing VPAC1-FLAG. The first method is based on an heat-shock protocol (16), in which the phage library was first incubated with WT CHO to remove non-specific binders. The subsequent supernatant was then incubated with CHO cells expressing VPAC1-FLAG. After thorough washing, cells were pelleted, resuspended in culture medium, and warmed at 37°C for 20 min. The remaining cell surface-bound phages were then stripped with 100 mM glycine buffer pH 2.5 containing 500 mM NaCl. The internalized phages were recovered by cell lysis with 100 mM TEA and amplified by infection of logarithmically growing *E. coli* TG1. The second biopanning method is a competition protocol. The phage library was incubated with CHO cells expressing VPAC1-FLAG. After thorough washing to remove non-specific binders, phages were eluted by 40 µM VIP and amplified by infection of logarithmically growing *E. coli* TG1. After two selection rounds, individual clones were picked and periplasmic extracts were prepared as described (15). The specificity of individual nanobodies was assessed by flow cytometry staining of VPAC1-FLAG overexpressing CHO cells and WT CHO cells. Unfixed, unpermeabilized cells were incubated with fivefold diluted periplasmic extracts containing the nanobody in FACS buffer (PBS, 10% FBS, 0.1% sodium azide) for 60 min at 4°C. After washing in FACS buffer, the bound nanobodies were detected with a FITC-conjugated anti-His Tag antibody (Bio-Rad; 1/50) and the median cell fluorescence (MCF) intensity was determined. The data were processed using the FACSDiva software (Becton Dickinson).

### Production and Purification of Anti-VPAC1 Nanobodies

The 12 unique nanobodies identified were expressed in the *E. coli* WK6Su^−^ strain and purified by metal affinity chromatography. Briefly, bacteria were grown in terrific broth supplemented with 100 µg/ml ampicillin, 0.1% glucose, and 2 mM MgCl_2_ to an OD_600_ of 0.6–0.9. The expression of nanobodies was then induced by adding 1 mM IPTG (Sigma-Aldrich) and bacteria grown overnight at 30°C. The bacteria were centrifuged at 7,000 *g* for 10 min, the pellets were resuspended in 15 ml of TES buffer (0.2 M Tris-HCl pH 8.0, 0.5 M sucrose, 0.5 M EDTA) and incubated for 1 h at 4°C under mild agitation. 30 ml of fourfold diluted TES buffer were added and the samples were further incubated for 45 min at 4°C under mild agitation. The samples were centrifuged at 8,000 *g* for 30 min at 4°C and the supernatants containing nanobodies were collected for purification on Ni-NTA resin (Thermo Fisher Scientific) (15). Binding of nanobodies on Ni-NTA resin was performed at room temperature for 1 h, the columns were then washed with 50 mM phosphate buffer pH 6.0, 1 M NaCl and nanobodies were eluted with 1 M NaCl in 50 mM sodium acetate buffer pH 4.5. The protein solution was neutralized by adding 1 M Tris-HCl buffer pH 7.5. Nanobody purity was verified by SDS-PAGE and quantity was evaluated by optical density measurement.

### FACS Analysis

Cells were detached from culture dishes using PBS containing 5 mM EDTA, harvested by centrifugation (560 *g*, 4°C, 3 min), and resuspended at a density of 10^6^ cells/ml in cold FACS buffer (PBS, 0.1% BSA, 0.1% sodium azide). The cell suspension (100 µl) was incubated with anti-human VPAC1 [(17); 1/100] or anti-VPAC2 [(11); 1/100] mouse mAbs, or nanobodies (300 nM). After 1 h of incubation at 4°C, the cells were washed with 2 ml cold FACS buffer and incubated for 30 min at 4°C in the dark with a FITC-conjugated anti-mouse IgG (Sigma-Aldrich; 1/100) for mAbs or with a FITC-conjugated anti-His Tag (Bio-Rad; 1/50) for unlabeled nanobodies. The cells were washed and resuspended in FACS buffer, and the fluorescence level was analyzed using an LSRFortessa flow cytometer (Becton Dickinson). The data were processed using the FACSDiva software (Becton Dickinson). Dead cells (forward scatter, 25) were excluded from the analysis, and nonspecific fluorescence was determined using untransfected cells or an APC-labeled control isotype.

### Nanobody Binding Assays

Purified nanobodies were fluorescently labeled with DyLight 650 according to the manufacturer’s instructions (Microscale Ab Labeling Kit; Thermo Fisher Scientific). Binding experiments were performed using DyLight 650-labeled nanobodies as tracers. For saturation experiments, VPAC1-FLAG overexpressing CHO cells were incubated for 1 h at 4°C with increasing concentrations of labeled nanobodies, in absence (total binding) or in presence of 3 µM unlabeled nanobody (nonspecific binding), in 100 µl cold FACS buffer. After addition of 2 ml cold FACS buffer, the cells were harvested by centrifugation (560 *g*, 4°C, 4 min), resuspended in cold FACS buffer and fluorescence was evaluated by FACS. Kd values were calculated by nonlinear regression of saturation curves using the GraphPad Prism software. For competition binding experiments, VPAC1-FLAG expressing CHO cells were incubated for 1 h at 4°C in 100 µl cold FACS buffer containing 10 nM DyLight 650-labeled nanobody and various concentrations of competitors [unlabeled nanobodies, VIP (Bachem), PACAP-27 (Bachem), PACAP-38 (Bachem)] or VPAC1 antagonist [Acetyl-(d-Phe^2^, Lys^15^, Arg^16^, Leu^27^)-VIP (1-7)-GRF (8-27), Bachem] (18). Following a washing step with 2 ml cold FACS buffer, the fluorescence of cells was evaluated by FACS. Non-specific binding was determined as the MCF obtained in the presence of 3 µM unlabeled nanobody, and IC_50_ values were calculated by nonlinear regression.

### [^125^I]-VIP Binding Studies

Binding studies were performed by using [^125^I]-VIP (PerkinElmer) as tracer on membranes prepared from VPAC1-FLAG expressing CHO cells as described previously (17). The non-specific binding was defined as the residual binding in the presence of 1 µM unlabeled VIP. Binding was performed for 30 min at 23°C in a total volume of 120 µl containing 20 mM Tris-maleate buffer pH 7.4, containing 2 mM MgCl_2_, 0.1 mg/ml bacitracin, and 1% bovine serum albumin. 3–30 µg of protein were used per assay. Bound and free radioactivity were separated by filtration through GF/B glass-fiber (Whatman) filters pre-soaked for 24 h in 0.01% polyethyleneimine and rinsed three times with ice cold 20 mM sodium phosphate buffer pH 7.4 containing 0.8% bovine serum albumin (Sigma-Aldrich).

### Cyclic AMP Production

cAMP accumulation in CHO cells expressing human VPAC1-FLAG receptor was measured using the cAMP dynamic two kit (Cisbio). Briefly, 2 × 10^4^ cells/well in 96-well plates were incubated for 15 min at 37°C—5% CO_2_ with 20 µl of ligands diluted in KRH/rolipram buffer (25 mM Hepes pH 7.6, 5 mM KCl, 1.25 mM MgSO_4_, 124 mM NaCl, 1.25 mM KH_2_PO_4_, 1.45 mM CaCl_2_, 0.1% glucose, 0.05% BSA, 25 µM rolipram). Cells were then lysed by adding 20 µl of lysis buffer (50 mM phosphate buffer pH 7.0, 625 mM KF, 0.75% Triton, 0.1% sodium azide) and the cAMP concentration was determined according to the manufacturer’s instructions. Basal activity was measured in presence of the KRH/rolipram buffer only.

### Human Leukocyte Populations

Leukocytes were isolated from venous blood of healthy donors by immunomagnetic bead cell sorting (MACS) according to the manufacturer’s specifications. Briefly, leukocytes were recovered on a Ficoll (Lymphoprep Axis-Schield) density gradient, and erythrocytes were lysed by ammonium chloride. Monocytes were purified by positive selection using CD14 microbeads (Miltenyi Biotec), resuspended at the density of 10^6^ cells/ml, and cultured in RPMI 1640 medium (Thermo Fisher Scientific) supplemented with 10% heat-inactivated FBS. Unbound leukocytes were recovered to purify T lymphocytes by negative selection using the Pan T Cell Isolation Kit (Milteny Biotec). T lymphocytes were cultured in RPMI 1640 medium supplemented with 20% heat-inactivated FBS, 1% sodium pyruvate (Thermo Fisher Scientific), and 1% non-essential amino acids (Thermo Fisher Scientific). Macrophages were differentiated from monocytes in the presence of recombinant human M-CSF (50 ng/ml, Peprotech) for 6–11 days. The purity of the cell preparations was evaluated by flow cytometry and over 85% of CD11b^+^/CD206^+^ macrophages were consistently obtained.

### Immunohistochemistry

Paraffin sections (5 µm) of normal tissue samples were obtained from CMMI-DIAPath (Belgium) and Biobanque Hôpital Erasme-ULB (BE_BERA1, Belgium). Sections were deparaffinized, rehydrated, microwaved for 20 min in citrate buffer (10 mM sodium citrate pH 6.0, 0.05% Tween 20) for antigen retrieval, and treated for 20 min with 3% hydrogen peroxide to block endogenous peroxidase. The sections were incubated for 30 min with 5% heat-inactivated normal serum and further with 300 nM of CA7281 nanobody diluted in PBS/1% heat-inactivated normal serum for 1 h at 4°C. After a short washing step, bound nanobodies were fixed by a 20 min treatment with 2% paraformaldehyde solution. Bound nanobodies were detected with successive 30 min incubations with a mouse anti-His Tag mAb (Bio-Rad; 1/2,000), a goat anti-mouse IgG antibody (Jackson; 1/100) and mouse peroxidase-antiperoxidase complex (Jackson; 1/400). After washing with PBS, sections were incubated with ImmPACT DAB Substrate (Vector Laboratories) and monitored under a microscope. Reaction was stopped by immersion of the slides in distilled water. The slides were counterstained with hematoxylin and eosine, dehydrated, coverslipped, and examined by light microscopy (Axioplan 2, Zeiss).

## Results

### Generation of Nanobodies That Bind to the Extracellular Domain of VPAC1

In order to generate nanobodies directed against the extracellular domain of human VPAC1, two llamas were immunized by DNA injections following a prime-boost strategy. Immune responses were primed with four doses of 2 mg endotoxin-free pCDneo plasmid encoding human VPAC1. A cell boost was performed, using VPAC1-FLAG overexpressing CHO cells saturated with 3 µM VIP, with the aim of favoring immunization against VIP-bound VPAC1. The mounting of the VPAC1-specific immune response was monitored by testing the sera of animals by FACS on CHO cells expressing the receptor. The two llamas showed VPAC1-specific seroconversion that slightly increased following the cell boost. After DNA immunization, and after the cell boost, PBLs were collected from the animals, RNA was prepared, and a phage library displaying the VHH repertoire was constructed.

A first round of panning was performed on VPAC1-expressing CHO cells using a heat shock protocol. This led to a clear enrichment in VPAC1-specific phages, but a second round of panning using the same approach did not improve the yield. After two rounds of panning on VPAC1-expressing CHO cells using a competition protocol, a clear enrichment of VPAC1-specific phages was detected. Two hundreds and seventy six clones resulting from panning with the heat shock protocol and 184 clones selected by panning with the competition protocol were further analyzed. A periplasmic extract was prepared from each clone and binding of the nanobodies to VPAC1 was assessed by flow cytometry, using VPAC1-FLAG overexpressing CHO cells, and WT CHO cells as negative control. Among all tested samples, 63 periplasmic extracts resulting from the heat shock panning protocol and 10 periplasmic extracts resulting from the competition panning protocol resulted in median cell fluorescence ratios ≥5 over WT CHO cells, and the corresponding nanobodies were sequenced. After sequencing, 12 unique nanobodies were identified that were classified into seven different families based on their CDR3 amino acid sequence and length.

### Specificity of Nanobodies and Epitope Mapping

The 12 nanobodies were purified by affinity chromatography on Ni-NTA columns, and their specificity confirmed by FACS analysis using WT and VPAC1-FLAG overexpressing CHO cells. All nanobodies gave greater median cell fluorescence values for VPAC1-FLAG than for WT CHO cells. However, we did not pursue the study of CA7287, CA7301, and CA7303, belonging to families 3, 5, and 6, respectively, as they displayed a high background signal on WT CHO cells (MCF ratio of 2:10 over secondary antibody alone).

For subsequent full characterization of the nanobodies, we focused on one member of each family: CA7277 for family 1, CA7281 for family 2, CA7293 for family 4, and CA7295 for family 7, and the four nanobodies were labeled with DyLight 650 fluorochrome (DL650).

**Figure 1:**
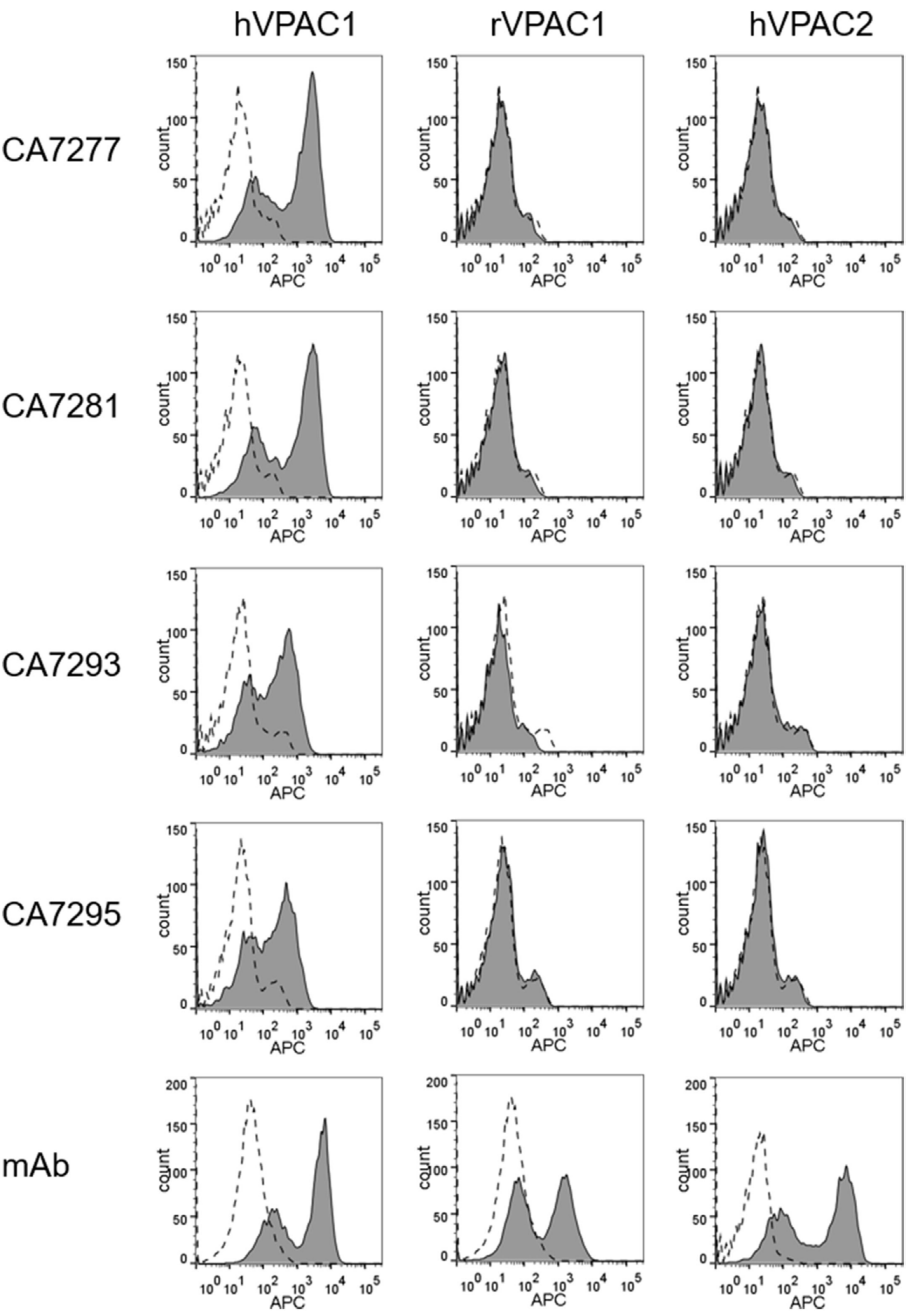
Assessment of nanobody specificity. The specificity of the nanobodies was investigated by FACS using human embryonic kidney cells-297T cells transiently transfected with empty plasmid (dotted lines) or plasmids encoding human VPAC1, rat VPAC1 or human VPAC2 (filled histograms). The cells were incubated with a reference mouse monoclonal antibodies (mAb) for each receptor (bottom panels) or DyLight 650-conjugated nanobodies. Bound mAbs was detected with an APC-conjugated secondary antibody. The data are representative of three independent experiments.

The specificity of the nanobodies was further explored by FACS analysis using HEK-293T cells transiently transfected with plasmids coding for human VPAC1, rat VPAC1, or the closely related human VPAC2 receptor. All nanobodies tested bound to HEK-293T cells expressing human VPAC1, but did not recognize rat VPAC1 nor human VPAC2 (Figure 1).

Taking advantage of their specificity for VPAC1, we grossly mapped the epitope recognized by the nanobodies, using FACS analysis and CHO cells stably expressing various VPAC1/VPAC2 chimeras. All four nanobodies bound to CHO cells expressing the chimeric receptor made of the *N*-terminus of VPAC1 and the core domain of VPAC2 (NT VPAC1/VPAC2), but did not recognize the chimeric receptor made of the *N*-terminus of VPAC2 and the core domain of VPAC1 (NT VPAC2/VPAC1), indicating that the *N*-terminal domain of VPAC1 is necessary and sufficient for nanobody binding (data not shown).

### Binding of Nanobodies to VPAC1

**Figure 2:**
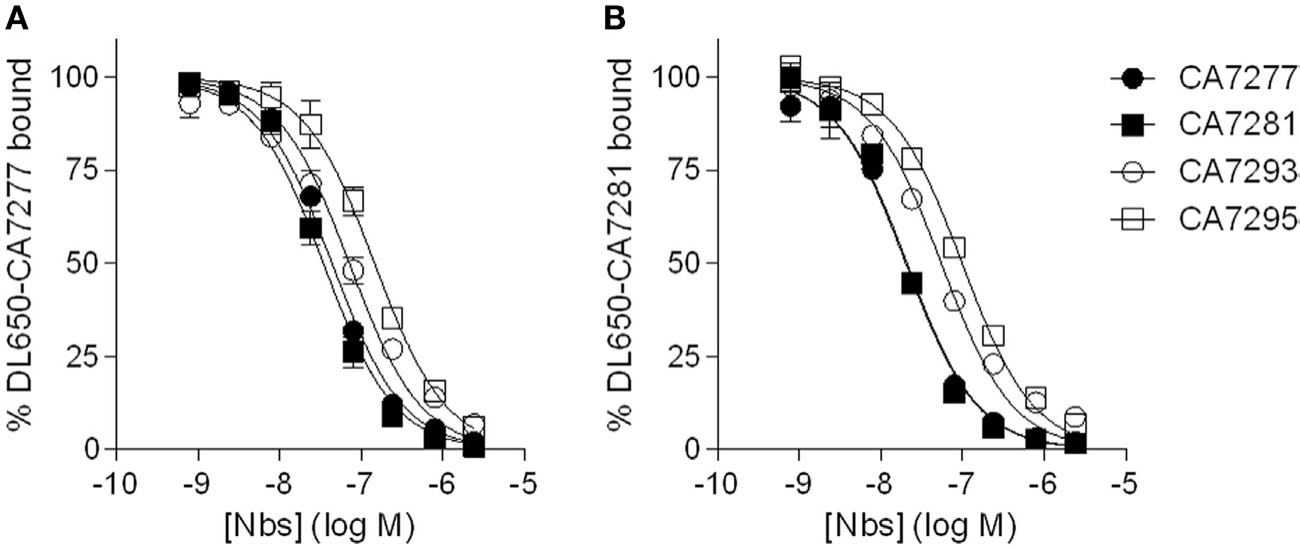
Nanobodies competition binding experiments. Binding of DyLight 650-conjugated CA7277 **(A)** and C7281 **(B)** on CHO cells expressing VPAC1 in presence of increasing concentrations of CA7277 (filled circles), CA7281 (filled squares), CA7293 (open circles), or CA7295 (open squares). The data represent the means ± SEM of three independent experiments performed in duplicate.

To evaluate the affinity of the nanobodies for VPAC1, we performed saturation binding experiments by flow cytometry using DyLight 650-labeled nanobodies as tracers. The median cell fluorescence intensity (MCF) of CHO cells expressing VPAC1 was recorded, following incubation with increasing concentrations of the tracer, in the absence (total binding) or presence of 3 µM unlabeled nanobody (nonspecific binding), and the Kd values were calculated from the saturation curves using non-linear regression. DL650-CA7277 and DL650-CA7281 bound VPAC1 with Kd values of 37 ± 8 nM and 26 ± 2 nM, respectively. For DL650-CA7293 and DL650-CA7295, we did not reach saturation for tracer concentrations up to 10 µM and the Kd values could not be calculated accurately (data not shown). We thus performed competition binding experiments using DyLight 650-labeled nanobodies to estimate the affinity of CA7293 and CA7295 and to evaluate whether the nanobodies recognize overlapping epitopes. We found that all nanobodies tested displaced DL650-CA7277 binding with IC_50_ values of 32 ± 3 nM (CA7277), 26 ± 3 nM (CA7281), 67 ± 13 nM (CA7293), and 117 ± 13 nM (CA7295). Similarly, CA7277, CA7281, CA7293, and CA7295 displaced DL650-CA7281 binding with IC_50_ values of 26 ± 3 nM, 21 ± 2 nM, 56 ± 4 nM, and 100 ± 5 nM, respectively (Figure 2). These data show that all nanobodies share a common, or largely overlapping, epitope. The IC_50_ values obtained from competition binding assays were similar to the Kd values derived from saturation binding curves for CA7277 and CA7281, suggesting that labeling of nanobodies with DL650 does not affect their interaction with VPAC1. As expected from the saturation binding experiments, CA7293 and CA7295 displayed higher IC_50_ values, indicating that they bind VPAC1 with lower affinity than CA7277 and CA7281.

### Effect of Nanobodies on VIP Binding

**Figure 3:**
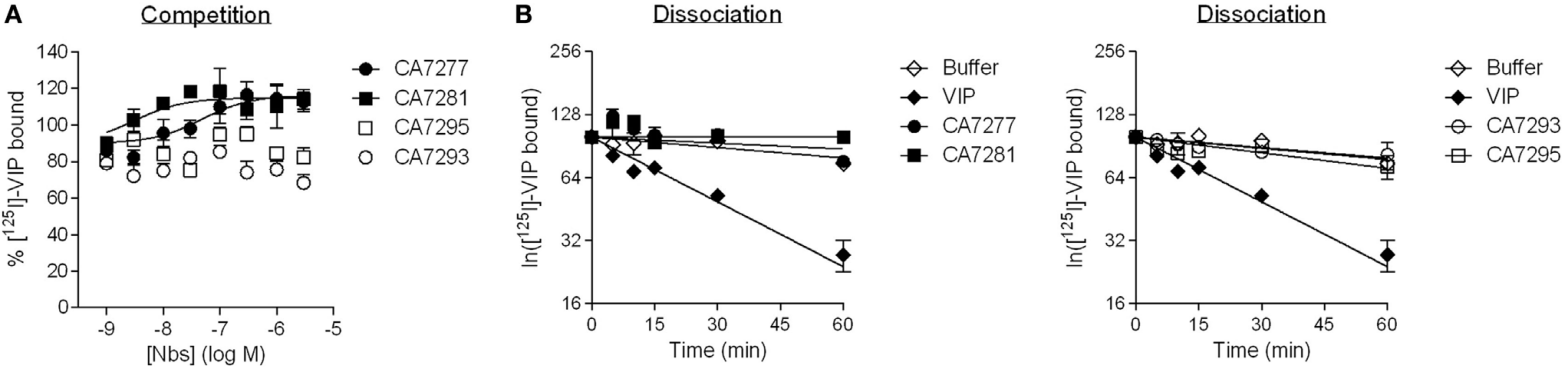
Vasoactive intestinal polypeptide (VIP) binding experiments. **(A)** Competition binding on CHO cells expressing VPAC1 using [^125^I]-VIP as tracer in presence of increasing concentrations of CA7277 (filled circles), CA7281 (filled squares), CA7293 (open circles), or CA7295 (open squares). **(B)** Kinetic studies of the dissociation of [^125^I]-VIP from CHO cells expressing VPAC1 (after 30 min of association at 23°C), following addition of 1 µM VIP (filled diamonds), 3 µM CA7277 (filled circles), CA7281 (filled squares), CA7293 (open circles), CA7295 (open squares), or buffer (open diamonds). The results are the means ± SEM of three independent experiments performed in duplicate.

We further tested whether the nanobodies were able to affect the binding of VIP, the natural ligand of VPAC1, on CHO cells expressing the human receptor. In a first set of experiments, we performed competition binding assays using [I^125^]-VIP as tracer. We found that CA7277 and CA7281 did not compete for the tracer, but instead dose-dependently increased, by up to 25%, its specific binding. CA7293 or CA7295 did not modify the binding of [I^125^]-VIP (Figure 3A).

As the increase of total specific binding of [I^125^]-VIP may reflect an increase of its affinity for nanobody-bound VPAC1, we next performed kinetic studies of radioligand dissociation. Addition, at steady state, of 1 µM VIP induced a rapid dissociation of the tracer that fits to a single exponential decay with a k*_off_* of 0.02 min^−1^ corresponding to a t_1/2_ of 29 min. In the presence of 3 µM nanobodies, the dissociation of the tracer was very slow and comparable to the kinetics observed in the presence of buffer only, with an estimated t_1/2_ of more than 120 min (Figure 3B). Together the results thus suggest that the epitope recognized by the nanobodies is distinct from the orthosteric binding site of VPAC1. The increase of [I^125^]-VIP binding in presence of CA7277 and CA7281 observed in competition binding experiments cannot be explained by a change in VIP affinity.

### Effect of VPAC1 Ligands on Nanobodies Binding

**Figure 4:**
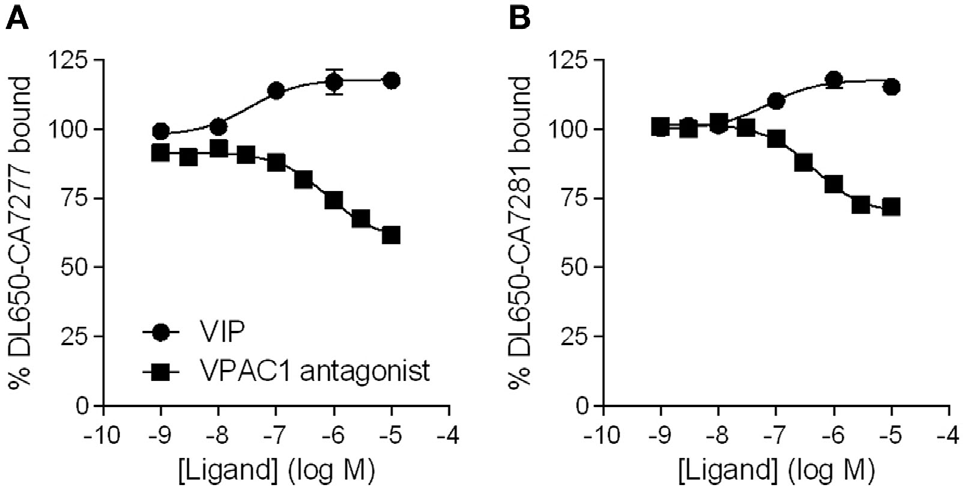
Effect of VPAC1 ligands on nanobodies binding. Binding of DyLight 650-conjugated CA7277 **(A)** and C7281 **(B)** on CHO cells expressing VPAC1 in presence of increasing concentrations of VIP (filled circles) or VPAC1 antagonist (filled squares). The results are the means ± SEM of three independent experiments performed in duplicate.

As we observed that CA7277 and CA7281 increase VIP binding, we wondered if VPAC1 ligand could also modify the binding of the nanobodies to VPAC1. We thus evaluated the effect of VIP and of a VPAC1 antagonist [Acetyl-(D-Phe^2^, Lys^15^, Arg^16^, Leu^27^)-VIP (1–7)-GRF (8–27)] (18) in binding experiments using DL650-CA7277 and DL650-CA7281 as tracers. Analysis of competition binding curves indicate that VIP dose-dependently increases by up to 20% the specific binding of the tracers, while the VPAC1 antagonist dose-dependently reduces by up to 40% DL650-CA7277 and DL650-CA7281 binding (Figure 4). As VIP and the VPAC1 antagonist share an overall common binding site, which is different from that of nanobodies, it is thus likely that the changes in DL650-CA7277 and DL650-CA7281 binding observed in presence of VIP and VPAC1 antagonist reflect changes in nanobody affinity. To verify this hypothesis, we performed saturation binding experiments in the presence of VIP or the high affinity-specific VPAC1 antagonist using DL650-CA7277 and DL650-CA7281 as tracers. Consistent with the competition binding studies, we observed that addition of 1 µM VIP significantly reduced the Kd value of CA7277 (Kd = 22 ± 5 nM) and CA7281 (Kd = 18 ± 2 nM), as compared to control conditions (Kd value of 37 ± 8 and 26 ± 2 nM, respectively). In contrast, addition of 1 µM VPAC1 antagonist increased the Kd value of CA7277 (Kd = 68 ± 7 nM) and CA7281 (Kd = 32 ± 5 nM).

### Effect of Nanobodies on VPAC1 Activity

**Figure 5:**
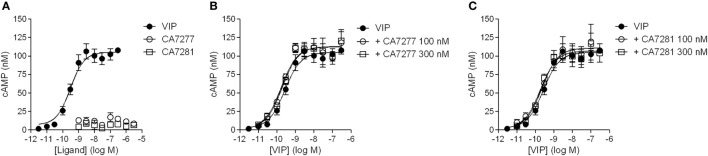
Effect of nanobodies on cAMP accumulation. **(A)** Cyclic AMP accumulation in the presence of VIP (filled circles), CA7277 (open circles), or CA7281 (open squares) in CHO cells expressing VPAC1 (left panel). Effect of CA7277 **(B)** or CA7281 **(C)** at 100 or 300 nM on cAMP accumulation promoted by increasing concentrations of VIP in the same cells. The results are the means ± SEM of three independent experiments performed in duplicate.

Cell-based signaling assays were performed to evaluate whether the nanobodies were able to modulate the activity of VPAC1. Incubation of CHO cells expressing VPAC1 with 300 nM of nanobodies did not induce cAMP accumulation over basal levels. Similarly, the four nanobodies tested did not modify the cAMP production promoted by 0.3 nM VIP. Finally, we evaluated the effect of CA7277 and CA7281 on dose-response curves of VIP-induced cAMP accumulation. As shown in Figure 5, the two nanobodies did not modify the potency or efficacy of VIP in this assay.

### Effect of Nanobodies on PACAP-Bound VPAC1

**Figure 6:**
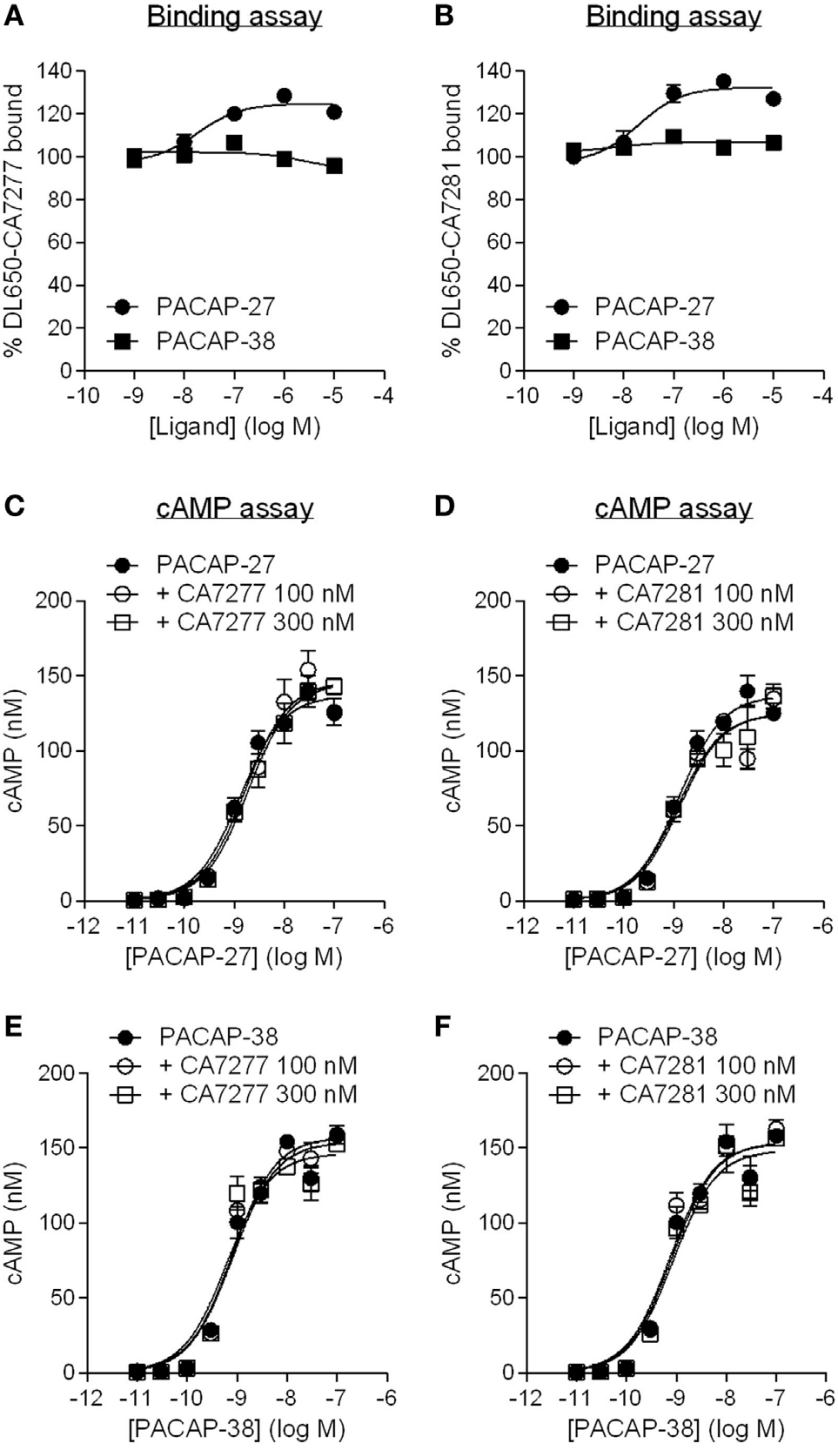
Effect of nanobodies on pituitary adenylate cyclase-activating polypeptide (PACAP)-bound VPAC1. Binding of DyLight 650-conjugated CA7277 **(A)** and C7281 **(B)** on CHO cells expressing VPAC1 in presence of increasing concentrations of PACAP-27 (filled circles) or PACAP-38 (filled squares). Effect of CA7277 **(C,E)** or CA7281 **(D,F)** at 100 or 300 nM on cAMP accumulation promoted by increasing concentrations of PACAP-27 **(C,D)** or PACAP-38 **(E,F)** in CHO cells expressing VPAC1. The results are the means ± SEM of three independent experiments performed in duplicate.

Although similar, many structural and pharmacological studies indicate that VIP and PACAP binding sites on VPAC1 slightly differ. This is particularly true for PACAP-38 which displays a 10 and 11 amino acids *C*-terminal additional sequence as compared to VIP and PACAP-27, respectively (2, 19, 20). Therefore, we also evaluated the behavior of CA7277 and CA7281 toward PACAP-bound VPAC1. As seen with VIP, PACAP-27 dose-dependently increases by up to 30% the binding of DL650-CA7277 and DL650-CA7281, while no effect was observed in presence of PACAP-38 (Figure 6). We were also unable to detect any activity of the two nanobodies in response to PACAP-27 or PACAP-38-induced cAMP accumulation (Figure 6).

### Immunodetection of VPAC1 on Human Primary Cells Using Fluorescently Labeled Nanobodies

**Figure 7:**
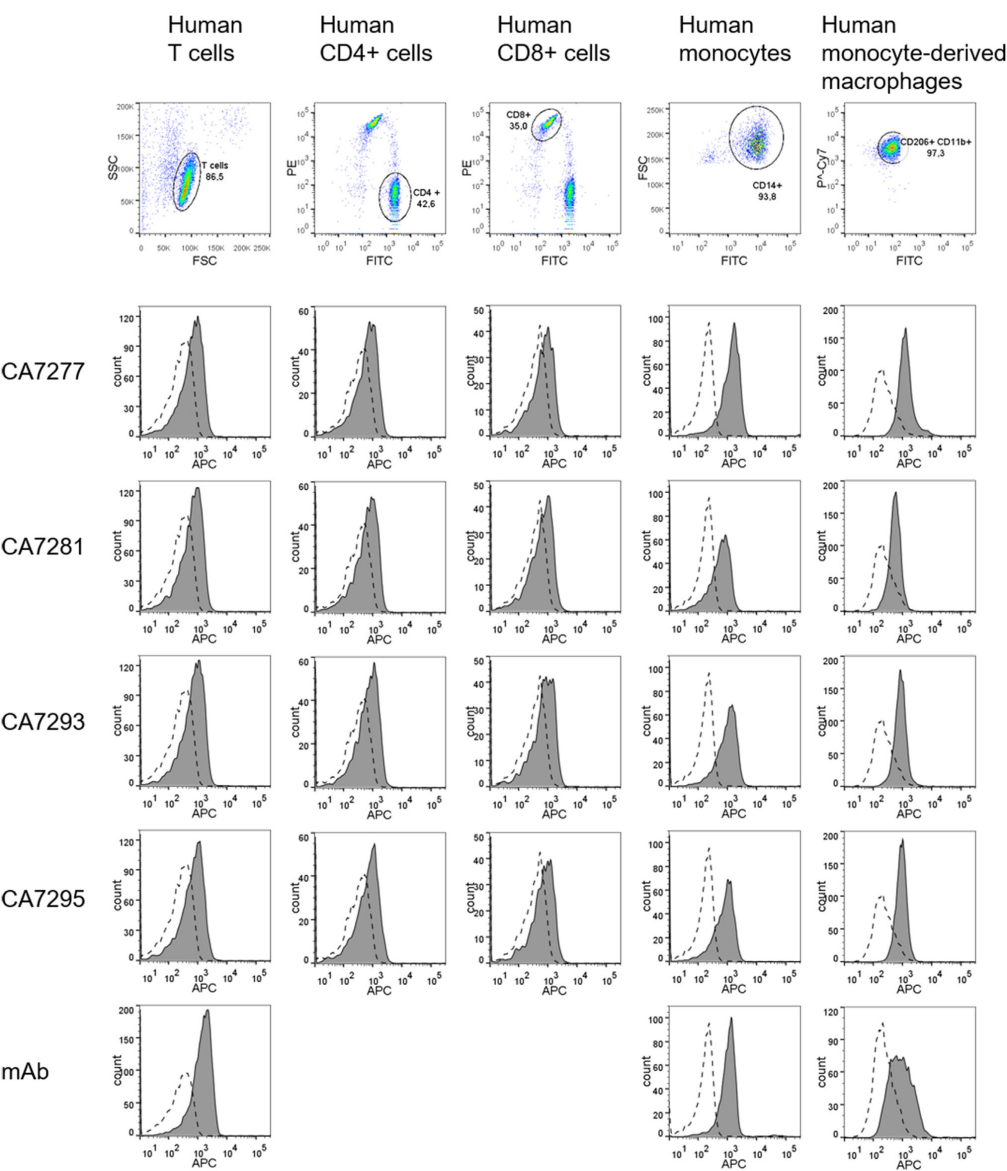
Immunodetection of VPAC1 on human primary cells. Cells were stained for leukocyte markers (CD3, CD4, CD8, CD14, CD11b, and CD206) and VPAC1 labeling was investigated on human T cells (first column), CD4^+^ T cells (second column), CD8^+^ T cells (third column), monocytes (fourth column), and monocyte-derived macrophages (fifth column), using a reference anti-VPAC1 mouse monoclonal (mAb) and DyLight 650-conjugated nanobodies. The background signal (dotted lines) was evaluated using a control isotype labeled with APC, or a DyLight 650-conjugated irrelevant nanobody. The data are representative of three independent experiments.

The ability of the nanobodies to detect VPAC1 on human primary cells was tested. Leukocytes were isolated from venous blood of healthy donors, T lymphocytes, and monocytes were isolated by immunomagnetic bead cell sorting and macrophages were derived from monocytes using standard culture conditions. The purity of the cell preparations was evaluated by flow cytometry using specific cell surface markers of leukocyte population. As shown in Figure 7, over 85% of CD3^+^ T cells (among which around 40% of CD4^+^ T cells and 35% of CD8^+^ T cells), over 90% of CD14^+^ monocytes, and over 95% of CD11b^+^/CD206^+^ macrophages were obtained. Expression of VPAC1 was monitored by FACS on each leukocyte population, by using a reference anti-VPAC1 mouse monoclonal as well as DyLight 650-conjugated CA7277, CA7281, CA7293, and CA7295 nanobodies. As shown in Figure 7, labeling of VPAC1 on the surface of human leukocytes was as efficient with the fluorescently labeled nanobodies as with the reference mouse monoclonal antibody.

### Immunodetection of VPAC1 in Normal Gastrointestinal Human Tissues

**Figure 8:**
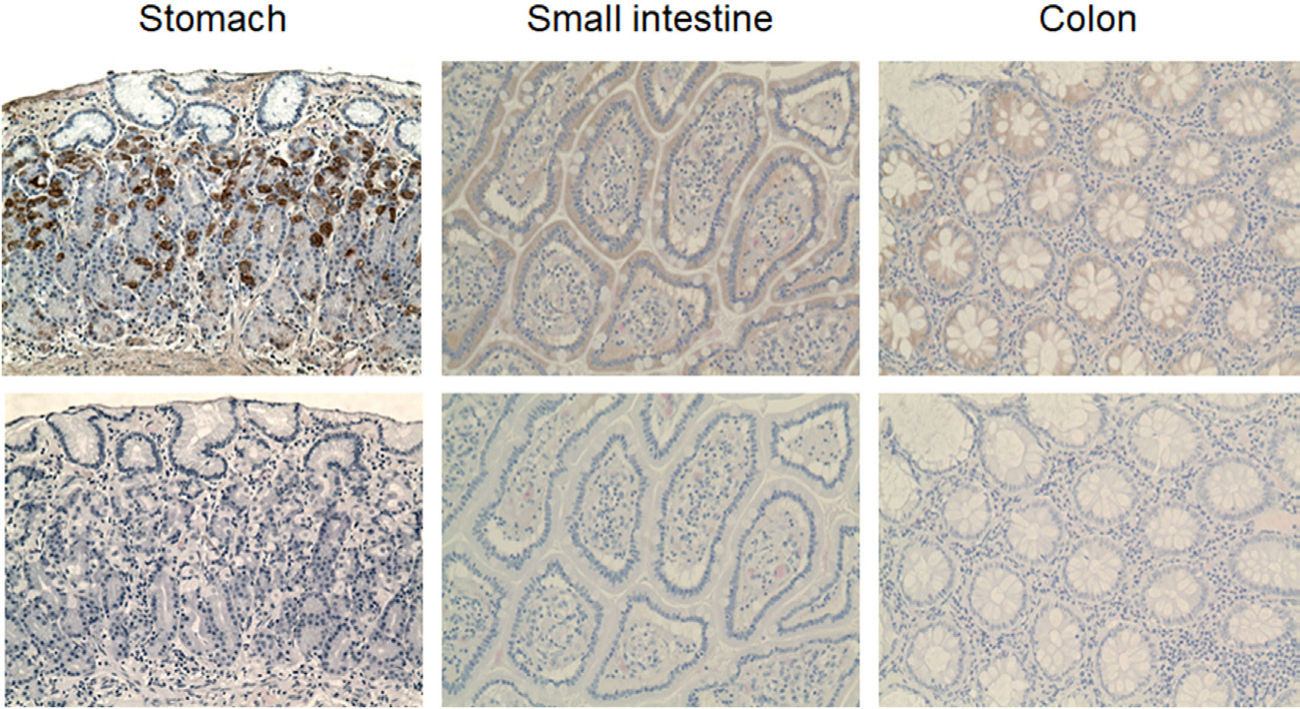
VPAC1 immunohistochemical staining in the normal human gastrointestinal tract. Sections of human stomach (left panels), small intestine (middle panels), and colon (right panels) were dewaxed, microwaved in citrate buffer, and incubated with 300 nM CA7281 (top panels). Bound nanobodies were detected using the peroxidase-antiperoxidase (PAP) method and sections were counterstained with hematoxylin and eosine. Bottom panels show the results obtained on adjacent sections treated similarly, but omitting the nanobody. Pictures were taken on a Zeiss Axioplan two microscope (original magnification 20×).

The nanobodies were also evaluated for immunodetection of VPAC1 on normal human tissue sections. Preliminary assays showed that heat-induced epitope retrieval is required for efficient immunological staining of paraffin-embedded tissue sections. Among the different methods tested, the citrate buffer heat-induced epitope retrieval was the more efficient (data not shown). We first used the DyLight 488-conjugated nanobody to perform immunofluorescence staining of VPAC1. Only DyLight 488-conjugated CA7277 and CA7281 nanobodies gave a detectable, although limited, signal. However, when compared to slides incubated with a control IgG labeled with FITC, the signal over noise ratio was too weak to validate unambiguous the labeling (data not shown). We thus decided to switch to immunohistochemical staining of the sections, a method offering a wide panel of signal amplification tools. In set up experiments, we found that the peroxidase–antiperoxidase method (PAP) was the most efficient to label VPAC1 with the nanobodies. We also observed that the signal obtained with CA7281 was more robust than that of CA7277 (data not shown). VPAC1 is known to be widely expressed in the body and particularly in the gastrointestinal tract. Thus to validate the ability of the nanobodies to detect VPAC1 in human tissue samples, we performed immunohistochemical staining of stomach and gut sections with the CA7281 nanobody. As shown in Figure 8, VPAC1 immunoreactivity was abundant in the mucosa of the stomach and intestine. In the stomach, CA7281 labeled some cells of the gastric mucosal glands. In small intestine (jejunum) and colon, we observed a clear signal in the absorbent epithelial cells.

## Discussion

The superfamily of GPCRs represents a large and diverse family of seven transmembrane domain proteins. Because of the receptor diversity and their involvement in all physiological processes, 30–40% of marketed drugs target GPCRs. However, current GPCR targeted drugs address only a small fraction of the GPCR repertoire (21). Therefore, GPCRs represent a major focus in functional genomics and drug development research with the ultimate aim of discovering novel drugs with high selectivity in their mechanism of action. Yet, in the past decade, the number of new small molecules targeting GPCRs that were approved as therapeutics has been limited. If some candidates reached preclinical and clinical trials, most studies were discontinued due to toxicity, poor efficacy, or inadequate selectivity (22, 23).

In the meantime, nanobodies have generated a growing interest as new research, diagnostic and therapeutic tools, because of their potential advantages over conventional antibodies. Multi-membrane spanning proteins, such as GPCRs and ion channels, are extremely difficult to purify as native proteins. Consequently, the generation of antibodies that recognize their clinically relevant conformation can be challenging. Following a procedure combining genetic immunization, phage display and biopanning on cells, we identified a panel of nanobodies targeting VPAC1. Nine nanobodies were developed, which specifically recognize human VPAC1, but not the closely related human VPAC2. They bind VPAC1 with moderate affinity and share a common binding site, as they competed with each other for binding. We also found that the epitope recognized by the nanobodies, localized in the extracellular *N*-terminal domain of the receptor, is distinct from the orthosteric binding site as nanobodies did not compete for VIP binding and *vice versa*. However, we observed that the affinity of two nanobodies (CA7277 and CA7281) was modified in the presence of VPAC1 ligands. Although slightly, their Kd values decreased significantly in the presence of VIP, while the Kd of CA7277 significantly increased in the presence of a VPAC1 antagonist. CA7277 and CA7281 exhibit, therefore, a higher affinity for the active receptor conformation, and they might, therefore, behave as allosteric modulators of VPAC1, with positive cooperativity toward VIP, but negative cooperativity toward the VPAC1 antagonist. However, they recognize as efficiently as a reference mouse monoclonal antibody an inactive VPAC1 receptor mutant (24), transiently expressed in HEK cells, which is unable to promote cAMP accumulation or calcium release in response to VIP (data not shown). Similarly, PACAP-27, but not PACAP-38 slightly increased the binding of the two nanobodies. It is, therefore, likely that CA7277 and CA7281 behave as very weak allosteric modulators of VPAC1, detectable only in some sensitive settings, but not in others. Indeed, no evidence of allosteric activity was seen in VIP binding studies, or in functional assays in response to VIP or PACAP.

Nanobodies are valuable tools in human medicine and have much to offer as a research tool because they are easy and inexpensive to produce at a laboratory level. Because they are encoded by a single gene of approximately 350 bp, nanobodies are very modular and can be easily modified and engineered to improve their properties and expand their usefulness in a broad range of applications. The first clinical trial involving a nanobody (ALX-0081), directed against von Willebrand factor, was launched in 2007. Although the development of ALX-0081 as an antithrombotic agent was discontinued, many other nanobodies are currently in the pipeline for the development of new treatments against inflammatory diseases and cancer (25). Interestingly, VPAC1 receptor is a promising target for the development of therapeutic molecules in such pathologies. Indeed, VIP, through VPAC1 activation, was shown to switch the Th1/Th2 balance in favor of Th2 immunity, and contribute to the generation and/or activation of regulatory T cells (26). In addition, VIP induces an inhibitory effect on innate immunity, by inhibiting the production of pro-inflammatory cytokines and chemokines secreted by macrophages (27). Numerous studies have also demonstrated that VPAC1 is overexpressed, resulting in high densities, in numerous cancers, including bladder, breast, colon, liver, lung, neuroendocrine, pancreatic, prostate, thyroid, and uterus cancers (28). Thus, our nanobodies may constitute innovative tools in these fields as we showed that they are able to detect clinically relevant native VPAC1 in human leukocytes and gastrointestinal tissues.

As they do not display functional properties and, like all nanobodies, they are devoid of Fc-domain, their use as pure immunotherapeutic is limited. However, they could be linked to effector moieties, such as drugs and macromolecules, to specifically target VPAC1-expressing cells. Similarly, they could be chemically attached to the surface of drug delivery systems. These two approaches have been already successfully applied to other nanobodies, such as those targeting VEGFR2 or EGFR, as well as to VIP derivatives to induce tumor cell death (25, 28). With the aim of targeting VPAC1, the use of nanobodies instead of VIP would improve specificity and efficiency, as they bind only one receptor population (VPAC1 and not the closely related VPAC2) and they are not subjected to enzymatic degradation like VIP.

The use of radiolabeled molecules combined to imaging techniques, such as single photon emission computed tomography (SPECT) or positron emission tomography (PET) is a technique that enables early detection of tumor cells, monitoring of disease progression and response to therapy. For example, the overexpression of somatostatin receptors (SSTR1-5) by neuroendocrine tumors is currently widely used, and when combined with SPECT is the most sensitive localization method so far. A similar approach is being investigated for a number of tumors overexpressing VPAC1 (mainly breast and prostate cancer) using various radiolabeled VIP analogs (28). Thus, as described for nanobodies targeting HER2 (25), our VPAC1 nanobodies labeled with radionuclides could constitute a new tool for clinical imaging. Besides their high specificity for VPAC1, the fact that they lack functional properties makes them particularly good candidates, as it will prevent adverse pharmacological effects during imaging.

As already mentioned, nanobodies constitute also valuable tools in diverse aspects of fundamental research. One of the major advantages of nanobodies as research tools is that they are easily customizable by engineering, allowing expanding their use in many applications. We and others confirmed the usefulness of nanobodies as equivalent surrogates for conventional antibodies (9, 29, 30), as shown in the current study with the detection of native VPAC1 in human leukocytes by FACS analysis using fluorescently labeled nanobodies. Fluorescent nanobodies are also efficient as primary detection reagents in fluorescent microscopy and live imaging on cells and whole organisms (9, 29). Recently, their use as nanoscale detection tools for high-resolution microscopy has emerged. Since nanobodies are significantly smaller, it has been possible to achieve resolutions of 25 nm, twofolds higher than what was achieved with indirect immunochemistry using conventional antibodies (31).

Nanobodies can be used for classical biochemical studies such as protein purification and study of protein-protein interactions using different strategies, such as fluorescent-three-hybrid system, affinity-purification mass spectrometry, or fluorescence resonance energy transfer (9, 29).

Engineered nanobodies have also been developed to investigate protein function. For example, it is possible to label nanobodies with a delocalization tag that will redirect the antigen-nanobody complex to predetermined organelles and consequently induce a loss of function. Other groups exploit the universal ubiquitin proteasome pathway in combination with high affinity nanobodies to induce targeted degradation of specific proteins, leading also to a loss of function (29).

The unique properties of nanobodies have stimulated their use in a broad range of applications and offer multiple opportunities as therapeutics and in fundamental research. In this study, we generated and characterized a panel of nanobodies recognizing human VPAC1, which constitute new original tools to further investigate the role of VPAC1 in physiological and pathological conditions.

## Ethics Statement

The experiments using human samples were carried out in strict accordance with the national and international guidelines in use at the Université libre de Bruxelles and in accordance with the Helsinki Declaration. All procedures were reviewed and approved by the local ethic committee (Comité d’Ethique hospitalo-facultaire Erasme-ULB [021/046]) of the Université libre de Bruxelles (Protocol approval reference P2017/197).

## Author Contributions

XP performed experiments, analyzed and interpreted data, and participated in writing. TL established experimental procedures, performed experiments, analyzed and interpreted data for generation of VPAC1 nanobodies, and participated in writing. VL, MD, and HH performed experiments and analyzed data. JS supervised the generation of VPAC1 nanobodies. MP participated in data interpretation and writing. IL initiated the study, established experimental procedures for characterization of VPAC1 nanobodies, coordinated the project, analyzed and interpreted data, and cowrote the manuscript. All authors reviewed and approved the manuscript.

## Conflict of Interest Statement

TL, MD, and HH are currently employees of Confo Therapeutics. The other authors report no conflict of interest.
